# 同时性多原发肺癌的诊治体会及处理策略新进展

**DOI:** 10.3779/j.issn.1009-3419.2018.03.11

**Published:** 2018-03-20

**Authors:** 连奎 韩, 树庚 高, 锋维 谭, 自然 赵, 朋 宋

**Affiliations:** 1 550000 贵阳，贵州省人民医院胸外科 Department of Toracic Surgery, Guizhou People's Hospital, Guiyang 550000, China; 2 100021 北京，中国医学科学院肿瘤医院胸外科 Department of Toracic Surgery, Tumor Hospital of the Chinese Academy of Medical Sciences, Beijing 100021, China

**Keywords:** 多原发肺癌, 胸腔镜, 外科手术, 薄层高分辨CT, Multiple primary lung cancer, Toracoscope, Surgery, Tin layer distinguish CT

## Abstract

**背景与目的:**

同时性多原发肺癌(synchronous multiple primary lung cancer, sMPLC)，既往属少见疾病，但近年来由于诊疗手段的进步检出率逐渐升高。但关于MPLC的发病机制、诊断、鉴别诊断和临床处理策略仍存在诸多争议。本研究对31例sMPLC的临床资料进行总结分析，进一步探讨sMPLC的诊断、治疗和预后。

**方法:**

归纳总结31例sMPLC的临床资料，回顾性分析其诊断方法、手术方式、病理资料。

**结果:**

所有患者均行胸腔镜手术，无死亡病例。术前均行薄层高分辨计算机断层扫描(computed tomography, CT)发现肺部多发结节。病灶位于同侧者均同期行手术治疗，手术方式以胸腔镜下肺叶+亚肺叶切除为主；病灶位于双侧者，均分期手术，时间间隔为3个月-4个月。

**结论:**

薄层高分辨CT是术前诊断sMPLC的最佳方法。sMPLC同侧肺发病率高于双侧肺(23:8)，周围型多见，占94%，组织学类型以腺癌最多见，占80.6%(25/31)。胸腔镜下主病灶的肺叶切除+次要病灶的亚肺叶切除是最常用的术式。

## Abstract (en)

**Background and objective:**

Simultaneity multiple primary lung cancer always is a rare disease, but in recent years due to the progress of the diagnosis and treatment means detection rate increased. Tis study summarized the clinical data of 31 cases of synchronous multiple primary lung cancer (sMPLC) analysis, further to explore the diagnosis, treatment and prognosis of MPLC.

**Methods:**

Sum up the clinical data of 31 cases of simultaneous multiple primary lung cancer, the diagnostic method, surgical methods, pathology, were retrospectively analyzed.

**Results:**

All the patients are thoracoscope surgery, no deaths. Tin high resolution computed tomography (CT) in preoperative found multiple lung nodules. Lesions located in the same side of the same period surgical treatment, the operation method is given priority to with under the thoracoscope lung + the lobectomy; Lesions located in bilateral, all staged operation, the time interval for 3 months-4 months.

**Conclusion:**

Tin layer distinguish CT preoperative diagnosis is the best way to simultaneous multiple primary lung cancer. Multiple primary lung cancer incidence of ipsilateral lung at the same time higher than that of bilateral lung (23:8), type, around 94%, the most common histology to adenocarcinoma, 80.6% (25/31). Primary lesions under thoracoscope lobectomy plus the lobectomy of secondary lesions is the most commonly used.

中国的流行病学调查显示，肺癌在各种肿瘤中的发生率及死亡率均占据首位^[1]^。多原发肺癌(multiple primary lung cancer, MPLC)是指在同一患者肺内同时或先后发生两个或两个以上的原发性恶性肿瘤，若两个肺部恶性肿瘤的诊断间隔时间在6个月之内，则称同时性多原发肺癌(synchronous MPLC, sMPLC)。随着多层螺旋计算机断层扫描(computed tomography, CT)广泛应用于肺癌术前检查，肺结节包括sMPLC的病例数逐渐增多，本文回顾性分析2017年1月-2017年12月中国医学科学院肿瘤医院胸外科收治的经手术治疗并经术后病理确诊的31例同时性多原发肺癌，对患者的一般资料、临床特点、影像学特征、手术方式、术后病理进行回顾性分析和讨论，现报道如下。

## 1 资料和方法

### 1.1 患者一般资料

分析2017年1月-2017年12月在中国医学科学院肿瘤医院胸外科手术治疗并经术后病理确诊的同时性多原发肺癌的31例的患者临床及病理资料。31例患者中男性13例(41.9%)，女性18例(58.1%)；发病年龄32岁-65岁，中位发病年龄52岁；吸烟者12例(38.7%)，不吸烟者19例(61.3%)；肺癌家族史阳性8例(25.8%)；个人既往有恶性肿瘤病史4例(12.9%)，病例的筛选参考Martini和Melamed(M-M)于1975年建立的DPLC诊断标准^[1]^，包括：sMPLC：①病灶位于不同部位，互相独立；②组织学类型不同；③组织学类型相同时需满足以下条件：位于不同的肺段、肺叶或双侧肺，且起源于不同的原位癌，共同的淋巴引流部位无癌，诊断时无肺外转移。

## 2 结果

### 2.1 发病部位

31例患者中共发现肺部结节81个，病变位置同侧肺23例(74%)：其中相同肺叶6例，同侧不同肺叶17例；不同侧肺8例(26%)；周围型病灶78个(94%)，中央型者3个(6%)。

### 2.2 CT资料

周围型病灶形态呈圆形或类圆形占50.6% (41/81)，不规则形占49.4%(40/81)；边缘见毛刺占74.0%(60/81)，胸膜牵拉占43.2%(35/81)。组织学类型以腺癌最多见，占80.6%(25/31)，非腺癌组病灶均表现为实性结节。腺癌组以不规则形多见，非腺癌组分叶征多见。

### 2.3 组织学类型

组织病理类型相同者19例(61.2%)，其中腺癌-原位腺癌3例(1 5.7 %)，腺癌-腺癌1 3例(68.4%)，鳞癌-鳞癌3例(15.7%)。组织学类型不同者12例(31.6%)，腺癌-鳞癌5例(41.6%)，腺鳞癌-腺癌5例(41.6%)，腺癌-大细胞癌1例(8.3%)，腺癌-小细胞癌1例(8.3%)。根据腺癌的最新病理分型，将腺癌结节亚分型统计为，非典型腺瘤样增生2个，原位腺癌3个，微浸润腺癌2个，浸润性腺癌63个，其中附壁样生长方式为主的亚型14个，腺泡样生长方式为主的亚型40个，乳头状生长方式为主的亚型9个。

### 2.4 术式选择

本研究的31例患者均行胸腔镜手术治疗。其中同时性同侧多原发肺癌中均行同期手术。而同时性双侧多原发肺癌中则均行分期手术，手术间隔平均3个月。由于每例患者均存在多个病灶，根据每个病灶的大小、密度、术前影像学良恶性的预估、术中冰冻病理诊断，综合考虑其他病灶的情况和患者的一般情况，采取了不同的术式。包括肺叶切除、楔形切除、肺段切除等。病变位于相同肺叶时采用肺叶切除+淋巴结清扫，共6例(19.4%)。病变位于不同肺叶时，采用肺叶+亚肺叶切除(包括肺叶切除+楔形切除、肺叶切除+肺段切除、肺叶切除+楔形切除+楔形切除) +淋巴结清扫者共21例(67.7%)，各病灶均行亚肺叶切除(包括楔形切除+楔形切除、肺段切除+楔形切除、肺段切除+肺段切除)者共4例(12.9%)。所有病例均行胸腔镜手术治疗，无中转开胸，无手术死亡病例。

## 3 讨论

随着年代的不同，以非小细胞肺癌为主的肺癌的临床特征及诊疗方式已经发生了巨大的变化，表现为从"中心型"病变向"周围型"病变的转变、鳞癌向腺癌的转变、单发病灶向多发病灶的转变。肺既是容易发生原发癌，也是容易发生转移的器官，在诊断sMPLC的过程中，先要排除肺转移的情况：一种是一个为原发灶其他为转移灶；另一种是原发灶在肺外器官，肺内病灶均为转移瘤。因为sMPLC与肺转移癌的临床治疗及其预后完全不同。后者基本无手术机会，远期生存率低。而sMPLC大多为早期癌，术后5年生存率较高。多原发肺癌，最初由Beyreuther^[2]^在1994年提出，以往认为sMPLC是一种较少见的疾病，但据近些年国内外的研究报道，其发病率呈不断上升趋势^[3, 4]^。因而sMPLC成为胸外科临床医师必须面对的问题，MPLC的诊断和治疗，特别是sMPLC的诊断和治疗，尚有一定难度，因此，对该类疾病的研究具有重要的临床价值。

MPLC发病率和检出率在不断提高，可能与以下因素有关：①随着国人生活水平的不断提高，医疗水平及就诊体检意识的增强, 检测手段如纤支镜、CT、正电子发射型计算机断层显像(positron emission tomography-computed tomography, PET-CT)等的普及应用；此外电视胸腔镜、纵隔镜等先进的胸外科微创技术的广泛开展，使以前有肿瘤手术病史，尤其是有胸部手术史或心肺功能等一般条件不能耐受常规开胸探查术的患者也得到了确诊的机会，其多原发癌的检出率也在不断提高。②随着医疗卫生水平的提高，人的平均寿命得以延长，与此同时也为肿瘤的发生发展提供了时间长度。随着肺癌早期检出率的提高，微创胸外科的发展以及新化疗药物的出现、多维放疗的应用，提高了初发癌患者的疗效，一方面这些措施使肿瘤患者生存期有所延长，另一方面同时也使多原发癌中的肺癌的发生几率有所增大。有文献报道恶性肿瘤患者在生存期超过3年以上，发生重复癌的概率是10%-25%^[5]^。初发癌术后化/放疗的应用，使本身免疫力就较低的肿瘤患者的肿瘤防御能力更为降低，已有文献报道化/放疗本身也具有远期致癌的可能^[6]^。③每个原发肿瘤都有其自身发展过程，虽是"同时"发现，但可能处于不同的发展阶段，只是同时发现而已，其组织学类型也可能会有所不同。恶性肿瘤患者本身具有多发癌的倾向，在发病机制上多数学者支持"多中心性起源"的概念，有研究^[7]^证明已发生恶性肿瘤的个体再次发生新的原发肿瘤的危险是正常人的1.29倍，两者之间的关系有待进一步的研究。④Strong等^[8]^首次把MPLC的区域性癌化假说运用到呼吸系统疾病。这一学说认为，整个呼吸系统中，支气管肺泡上皮广泛异型增生，可能与致癌原介导的DNA改变有关，某些高级别异型增生的细胞先癌变，随时间的推移，其他不同部位处于不同级别的异型增生细胞相继基因突变，形成所谓具有独立且不同克隆来源的多原发肺癌可能。另外临床医生对多原发癌的认识不断提高，当肿瘤病人再发现肿瘤时，不仅仅考虑转移的可能，也重视了多原发癌的诊断。

对于MPLC的诊断标准，20世纪多参照Martini-Melamed诊断标准^[9, 10]^。同时性肺内多原发性癌(synchronous MPLC, sMPLC)的诊断标准是：①双肺同时发生或在间隔6个月以内发生病变；②经病理诊断证实是恶性肿瘤，其组织学类型不同。组织学类型相同时：位于不同肺段、肺叶，不同侧肺，由不同的原位癌起源，肺癌共同的淋巴引流部位无癌肿，确立诊断时无肺外转移；③除外复发和转移。异时性多原发肺癌(metachronous MPLC, mMPLC)：①组织学类型不同；②组织学类型相同时：无瘤间期至少2年，或均由不同的原位癌起源，或第二原发癌位于不同肺叶或不同侧肺时，肺癌共同的淋巴引流部位无癌肿，确立诊断时无肺外转移。而各个肿瘤具有独特的病理形态特征为诊断MPLC的要点。随后研究^[11, 12]^相继对M-M标准进行了修订，主要的更改是将无瘤间期由2年延长为4年，各个修订版本都强调，相对于判断新发病灶是原发还是转移，更为重要的是判断其是否能接受治愈性的治疗方式。但是仅依靠上述标准诊断多原发病灶依旧存在一定的困难，缺乏分子生物学理论的支撑。进入21世纪后，美国胸科医师联盟(American College of Chest Physicians, ACCP)再次对M-M标准进行了修订，引入了分子基因诊断作为标准，提出利用不同的分子遗传学特点对相同病理类型的多发病灶加以鉴别^[13]^。

鉴于MPLC以肺腺癌居多，2013年ACCP推荐利用肺腺癌的组织学亚型鉴别MPLC与肺内多发转移，还提出了另外一种鉴别手段——分子遗传学分析，即利用特异的分子标志物或基因突变位点加以鉴别^[14]^。研究者们开始试图利用不同的分子遗传学指标来协助MPLC的诊断，基因拷贝数^[15]^、杂合性丢失(loss of heterozygosity, LOH)^[16]^、*EGFR*/*Kras*突变状态、微卫星标记等位基因变异等，但是上述研究的样本量均较小，且实施具有一定的技术难度，尚没有哪种方法得到广泛的认可。因此ACCP指南建议仍需综合考虑临床特点及影像学特征鉴别MPLC和肺内转移。也就是说目前尚无诊断MPLC的金标准，以上标准仅供参考。MPLC需要多学科综合诊断，包括:放射科、肿瘤内科、胸外科、病理科医师共同参与，如条件许可，可进行病灶活检。MPLC的诊断需考虑以下因素：组织学类型、遗传学特点、影像学特征以及临床表现。

MPLC的影像学特点：PET-CT在发现肺部肿瘤淋巴结转移及远处转移方面有其独特的优势，但并非对所有的肺部小结节都有诊断价值。对同时性多原发肺癌的病灶，术后的病理类型多为腺癌，其中有很大一部分在影像学上表现为磨玻璃样结节。对于这一类不含实性成分的结节，通常很少有代谢增高及淋巴结转移，所以PET对多原发肺癌的诊断意义不大。多层螺旋CT具有很好的空间分辨力及密度分辨力，有利于观察结节内部及周围的细节征象，对鉴别诊断很有帮助。目前临床上术前对SMPLC的诊断及鉴别主要仍依赖于胸部CT，MPLC的各癌灶大多具有原发性肺癌的典型CT表现，多为孤立的，如边缘毛刺、胸膜牵拉、分叶、血管集束征、密度混杂(如磨玻璃样或实性)、增强窗可见强化等，并且肿瘤倍增时间较长。转移癌则常表现为边缘光滑、密度较均匀的球形阴影，少见毛刺及分叶征，多位于肺周围的浅表层，肿瘤倍增时间较短。另外，CT的随访观察也是对初次影像表现难以定性诊断者的重要方法，Lee等^[16]^研究认为良性病灶在短期内CT复查一般有明显的变化，除局灶性肺间质纤维化以外，良性病灶一般在3个月内部分吸收或完全吸收，原因在于良性病灶常是炎症、局灶性出血、肺水肿等，能够自发吸收或经抗炎或激素治疗后吸收。肿瘤或局灶性肺间质纤维化经过长期随访后可以长期存在，不会变小或吸收。若随访过程中，病灶增大、大小无变化但密度增高、内部出现实性成分或实质性成分增多，则提示恶性几率明显增大，需要及早手术。因此对于CT扫描检出的病灶，初诊不能定性的，严格的影像随访，密切观察病灶变化是十分必要的。sMPLC，即使是周围型较小的非实性结节、部分实性结节及实性结节，也大多具有典型原发肺癌的影像表现如边缘带毛刺、片状磨玻璃样影伴胸膜牵拉等。而肺单发或多发转移瘤多为圆形和椭圆形，边缘一般较光整，很少具有毛刺和胸膜牵拉征象，病情进展迅速，患者一般情况较差^[17]^。具有肺外原发肿瘤病史，特别是结直肠癌，骨骼软组织肉瘤等，肺内单发结节为肺转移瘤的几率增大，可达54.7%^[18]^，因此详细全面的病史对于正确诊断有重要价值。

目前，关于多原发肺癌的治疗还没有权威指南，但大多数肺癌中心的报道认为积极的手术切除依然是治疗多原发肺癌最有效的方法^[19]^，如Mun等^[18]^报道Ⅰ期sMPLC 5年生存率可高达75.8%，远高于晚期肺癌的生存率，故对肺多原发癌应尽量争取及时手术切除以求获得最佳疗效。目前的外科治疗原则：与单发肺癌一样，手术应遵循肿瘤外科治疗的两大基本原则，即最大限度地切除肿瘤组织，同时最大限度地保留正常肺组织。同时性多原发肺腺癌因涉及多个病灶的治疗策略是：①在无手术禁忌证的情况下尽可能手术治疗；②尽可能完整有效地切除肿瘤；③尽可能多地保留健康肺组织；④术后应采取多学科综合治疗，以提高生存率。外科术式的选择：(1)同一肺叶双原发或多原发：同期手术多采用肺叶切除；不同肺叶单发病灶：位于同侧不同肺叶单发病灶:若患者肺功能允可，可采取同期手术，一般较大病灶所在的肺叶行肺叶切除术，小病灶采取肺楔切；若两病灶较小，可采用不同肺叶局部切除。(2)当病灶分别位于两侧肺叶时，大多数情况选择分期进行切除。遵循的手术原则是：①先切除中央型、进展较快、病灶较大或伴有纵隔、肺门淋巴结转移的病灶，后切除周围型、进展较慢、病灶较小或无淋巴结转移的病灶。②先切除对预后影响较大的病灶：如病灶较大、密度较高、实性成分比重较大、恶性征象明显、分期较晚的病灶。③两次手术间隔时间太短不利于患者初次手术后的恢复，增加了二次手术的风险；而间隔时间太长则会增加未切除侧病灶播散和转移的风险，一般两次手术的时间间隔应在3个月-4个月。(3)关于淋巴结清扫：无论采取何种手术方式，系统的淋巴结清扫都是必要的，它对延长肿瘤局部控制时间、提高治愈率及完善诊断分期均具有重要意义。(4)目前的肿瘤-淋巴结-转移(tumor-nodemetastasis, TNM)分期系统并不能正确地反映MPLC的生存和预后。应该将多发病灶单独分期，分期较高者作为患者最终分期。Kocaturk等^[20]^研究表明根据肿瘤分期采取术后辅助放化疗预后较好，全肺切除患者预后较差。MPLC预后较肺内转移好，5年生存率高达30% -50%。对于MPLC患者，建议对病理标本做基因突变检测，为术后辅以靶向治疗提供应用指证。(5)双肺多发病灶：内科治疗为主，先剖胸探查获取病理及基因检测结果，根据*EGFR*突变状态选取治疗措施，如*EGFR*敏感突变，则选择靶向治疗或化疗，若为*EGFR*野生型则以化疗为主。

随着CT肺癌筛查项目的普及和对多原发肺癌认识的提高，多灶肺癌的检出率逐渐上升。通过分子遗传学检测将有助于多原发肺癌与肺内转移的鉴别，从而为多灶肺癌的诊断和分期及治疗做出贡献。目前，大多数肺癌中心的报道认为积极的手术切除依然是治疗多原发肺癌最有效的方法，可以预期，随着MPLC研究的广泛展开和不断深入，将会有更加合理规范的指南以引导临床实践。
